# Fiber Optofluidic Technology Based on Optical Force and Photothermal Effects

**DOI:** 10.3390/mi10080499

**Published:** 2019-07-26

**Authors:** Chenlin Zhang, Bingjie Xu, Chaoyang Gong, Jingtang Luo, Quanming Zhang, Yuan Gong

**Affiliations:** 1 Science and Technology on Security Communication Laboratory, Institute of Southwestern Communication, Chengdu 610041, China; 2 Key Laboratory of Optical Fiber Sensing and Communications (Ministry of Education), School of Information and Communication Engineering, University of Electronic Science and Technology of China, Chengdu 611731, China; 3 State Grid Sichuan Economic Research Institute, Chengdu 610041, China

**Keywords:** optofluidics, optical force, photothermal effect, optical manipulation, optical fiber sensors

## Abstract

Optofluidics is an exciting new area of study resulting from the fusion of microfluidics and photonics. It broadens the application and extends the functionality of microfluidics and has been extensively investigated in biocontrol, molecular diagnosis, material synthesis, and drug delivery. When light interacts with a microfluidic system, optical force and/or photothermal effects may occur due to the strong interaction between light and liquid. Such opto-physical effects can be used for optical manipulation and sensing due to their unique advantages over conventional microfluidics and photonics, including their simple fabrication process, flexible manipulation capability, compact configuration, and low cost. In this review, we summarize the latest progress in fiber optofluidic (FOF) technology based on optical force and photothermal effects in manipulation and sensing applications. Optical force can be used for optofluidic manipulation and sensing in two categories: stable single optical traps and stable combined optical traps. The photothermal effect can be applied to optofluidics based on two major structures: optical microfibers and optical fiber tips. The advantages and disadvantages of each FOF technology are also discussed.

## 1. Introduction

Optofluidics is a reconfigurable, sensitive, and portable technology that combines microfluidic systems and the optical systems [1]. Microfluidic technology makes an optofluidic system more reconfigurable because the liquid of microfluidics has a unique flexibility for solid materials. Optical technology can enhance the sensitivity of optofluidics by introducing new functionality and opto-physical effects and can work at very small sizes in microfluidic channels. As a result, with the integration of microfluidics and an optical system, optofluidics has become suitable for multiple applications, such as medical diagnosis and treatment, environmental analysis, and substance analysis [2].

**Figure 1:**
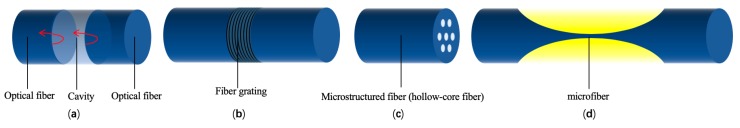
Fiber optofluidic (FOF) studies based on the structures of (**a**) the fiber-optic interferometer, (**b**) fiber grating, (**c**) microstructured optical fibers, and (**d**) optical microfibers.

Fiber optofluidic (FOF) technology is an important branch of optofluidics. As shown in Figure 1, FOF has four major types of structures, the fiber-optic interferometer, fiber grating, microstructured optical fibers (MOFs), and optical micro/nano fibers. In practice, FOFs show several notable superiorities [3,4]. FOF devices are inexpensive and simple thanks to the mature manufacturing technology of optical fibers. An FOF system can easily couple light into a chip with channels at a submillimeter scale because of the tiny cross-section of optical fibers and can accurately interact with liquid samples based on its low loss transmission. Additionally, an FOF system can improve performance with the notable fabricability of its optical fibers. For example, optical micro/nano fibers fabricated with commercial fibers can enhance the sensitivity of the environment through the evanescent field. Microstructured optical fibers (MOFs) can serve as both light transmission and microfluidic channels, due to their specific structures, such as a suspended core, a hollow core, and air cladding [5]. 

Fiber-optic interferometers can be divided into several major categories according to their structures, including a Fabry–Perot interferometer (FPI), whose interference is based on single arm incident laser oscillated in a cavity; and Mach-Zehnder, Michelson, and Sagnac, whose interference occurs via a two-arm optical laser split from the incident laser [6]. The typical structures of fiber-optic interferometers are summarized in Table 1.

A Fabry–Perot interferometer (FPI) can be used for optofluidic sensing based on either an extrinsic or intrinsic cavity, which is usually fabricated with two parallel mirrors, as shown in Figure 1a. The FPI can be designed for sensing with high performance by filling the special material or optimizing the mirrors of the cavity. A high-visibility in-line with the optofluidic Fabry–Perot cavity was demonstrated by splicing a silica capillary tube into two single mode fiber (SMFs) and polishing the latter optical fiber of the FPI [7]. Refractive index detection with a high sensitivity of 1148.93 nm/RIU was achieved thanks to the smooth end faces of the SMFs. Another sensing probe based on FPI was fabricated by sandwiching a cavity between the single mode fiber (SMF) facet and a Nafion film [8]. The results indicated that Nafion could be used for temperature and humidity sensing. 

Traditionally, two-arm interferometric structures, i.e., a Mach–Zehnder interferometer (MZI), a Michelson interferometer (MI), and a Sagnac interferometer (SI), are composed of two separate fibers [6]. Recent studies show that these two-arm interferometric structures can also be implemented as an in-line fiber optic core-cladding-mode interferometer, and thus have the advantage of compactness, simplicity, easy alignment, high coupling efficiency, high stability, and low-cost. Two-arm interferometric structures are promising for many sensing applications, such as label-free biosensing [9], humidity sensing [10,11], temperature-immune refractive index (RI) sensing [12,13], temperature sensing [14], and pressure sensing [15,16]. 

The optical fiber grating (OFG) is inscribed in the fiber core to form a sensing probe with the grating on the fiber tip or along the fiber axis. OFG can detect the changes of parameters in microfluids by mode coupling between the forward and backward transmission modes in the fiber core, or between the transmission mode in the core and in the cladding [17,18]. A sensing probe based on fiber Bragg grating inscribed in the photonic crystal fiber (PCF) was proposed for DNA detection, which is the first direct measurement of genomic DNA without a polymerase chain reaction (PCR) or other amplification reactions [17]. An in-line fiber optofluidic RI sensor was also proposed based on long-period fiber grating inscribed in a side-channel photonic crystal fiber [18]. A linear response and a sensitivity of 1145 nm/RIU was demonstrated.

Microstructured optical fibers (MOFs) are an excellent structure for optofluidic sensing due to their effective sample delivery and optical transmission. In [19], a side-channel photonic crystal fiber (PCF) was designed as a compact and ultrasensitive all-in-fiber optofluidic sensing platform. The large channel on one side of the fiber core enables a strong light-matter interaction and easy lateral access of liquid samples. It offers promising applications in chemical and biological analysis for monitoring the environment or biological/medical diagnosis. The working principle of optical micro/nano fiber sensors is based on an evanescent field. The electromagnetic field will partially penetrate into the cladding region to form an evanescent field, when the photon beam propagates through the core [20]. 

For the micro/nano fiber with a subwavelength diameter, the evanescent field is improved and interacts with the microfluid for sensing, as shown in Figure 1c. A microfiber, for which the diameter of the narrowed region is 7.8 μm, can achieve high humidity sensing [21]. A three-dimensional (3D) graphene network coated on the cladding of the microfiber can enhance the interaction between the moisture molecules and the three-dimensional graphene network (3-DGN) cladding. The relative humidity (RH) sensor displayed a fast response time of 4.0 s and an ultrahigh sensitivity of −4.118 dB/%RH in a relative humidity range from 79.5% RH to 85.0% RH.

Optofluidics not only structurally combine optics and microfluidics, but also induce new opto-physical effects from the energy transfer between light and microfluid and thus allows new schemes and functions for manipulation and sensing. However, few papers have comprehensively summarized the state-of-art fiber optofluidic (FOF) technologies in the literature. Recently, Vaiano et al. published an excellent review article about fiber optofluidics, which for the first time summarized the developments of the “lab on fiber (LOF)” concept for biological sensing applications [22]. They introduced the LOF technologies in three classes: lab on tip, lab around fiber, and lab in fiber, according to the integration location of functional unit. They compare the LOF technologies in terms of principle of operation, fabrication method, versatility in the design, and performance. They mainly focused on the functional material and structure of fibers in a microfluidic environment. The interaction between optics and microfluidics deserves another review to reflect its progress. 

In this review, we summarize the latest progresses in fiber optofluidic (FOF) technology and analyze the correlation between fiber optics and microfluidics. Specifically, we mainly consider the optical force and photothermal effect, because they have been studied extensively, and many applications of manipulation and sensing in optofluidics have been demonstrated. Other photo-physical effects, such as the photoacoustic effect, are beyond the scope of this review. 

The optical force can be generated by radiation pressure. It was first reported in 1970s, when Ashkin observed particle acceleration by a single laser beam [23]. Optical trapping with single laser beam was achieved in 1986 [24]. After that, researchers realized that optical force might be an effective tool for manipulating objects at a microscale [25,26,27,28]. Optical manipulation is a noncontact and nondestructive method and thus has great potential in the biological and healthcare sectors [17,25,26]. Additionally, optical force can be used for optofluidic sensing because its characters would be influenced by the trapped object or the microfluidic environment [29,30,31,32]. 

The photothermal effect may cause a thermal rise from laser beam, when liquid or objects in the microfluid absorb the laser energy. More specifically, the status of the target particle and/or the solvent may vary when the laser beam irradiates. The variations of charge carriers, molecular orientation, electrostriction, and radiation pressure in materials influence the conversion process of light energy to thermal energy. The changes in thermal energy further affect the temperature, refractive index, and the volume of the components in optofluidics, including liquid, objects, and the optical structure. In 1880, Bell and his coworkers reported the photothermal phenomenon in their paper on the photoacoustic effect [33], and the mechanism was reported by Terazima [34]. The photothermal effect has several unique advantages and can be used in optofluidics for multiple applications. First of all, the photothermal effect is applicable to a mix of solid, gas, and liquid matters states. Therefore, this effect is helpful to investigate the energy transfer between these states. Secondly, the photothermal effect has the potential for optofluidic multi-parameter detection and control. Lastly, the photothermal effect provides a non-contact method, which reduces the risk of mechanical damage in optofluidics.

The rest of this review is organized as follows. Section 2 introduces FOF technology based on optical force. The applications of optical trapping, manipulation, and sensing can be achieved with the stable single optical trap (SSOT) and stable combined optical trap (SCOT). The SSOT is formed only with optical force, while the SCOT is formed with the help of microfluidic flow force. Section 3 introduces FOF technology based on the photothermal effect. This device is mainly performed with two structures: the optical microfiber and optical fiber tip. 

## 2. FOF Technology Based on Optical Force

Conventionally, optical force was often generated based on light beams in free space focused by a high numerical aperture (NA) microscope objective, which is known as optical tweezers [24,35]. This bulky device makes optical tweezers difficult to use and expensive. The optical force based on the optical fiber structure offers many advantages over conventional methods, such as its low cost, compact configuration, easy integration, flexibility, and long transparent distance. It can serve as a versatile tool for optical manipulation and sensing. However, it is challenging to generate a stable trap for the optical fiber structure due to the low NA of the fiber. To resolve this problem, two main categories of optical traps (i.e., the stable single optical trap (SSOT) and stable combined optical trap (SCOT)) have been developed. The SSOT is formed only with the optical force, which is mainly based on the structures of the lensed fiber or fiber taper, dual-beam fiber trap, and special constructions. SCOT is formed with a balance of optical force and microfluidic flow force, which can provide more flexible manipulation with a longer distance.

### 2.1. Stable Single Optical Trap with Optical Force

**Figure 2:**
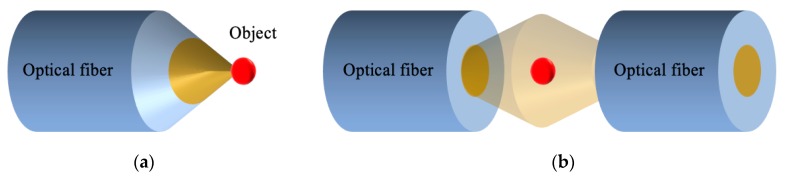
The schematic principle for stable single optical trap generation by (**a**) a fiber taper and (**b**) a dual-beam fiber trap.

A stable single optical trap (SSOT) can be formed only with optical force to achieve trapping and manipulation. Since the optical force generated by a flat fiber tip usually pushes the object away, SSOT either utilizes a single optical force formed by a lensed fiber or fiber taper to enhance convergence (see Figure 2a) or utilizes the dual-optical forces formed by dual beams from different directions to maintain balance (see Figure 2b).

#### 2.1.1. SSOT Based on Single Optical Force

A fiber taper is a straightforward way to form an SSOT by enhancing the trapping efficiency of a single optical fiber. The fiber taper can strongly focus the laser beam with a lens-like structure, allowing a 3D SSOT to be achieved [36]. This structure is easy to fabricate with a commercial optical fiber by chemical etching [37,38,39], polishing [40], or heating-and-drawing [36]. However, it has the limitation of a short working distance and fixed SSOT due to the firm, sharp structure of the fiber taper. The fiber taper can trap an object, but it hardly changes the object’s position without moving itself. 

**Figure 3:**
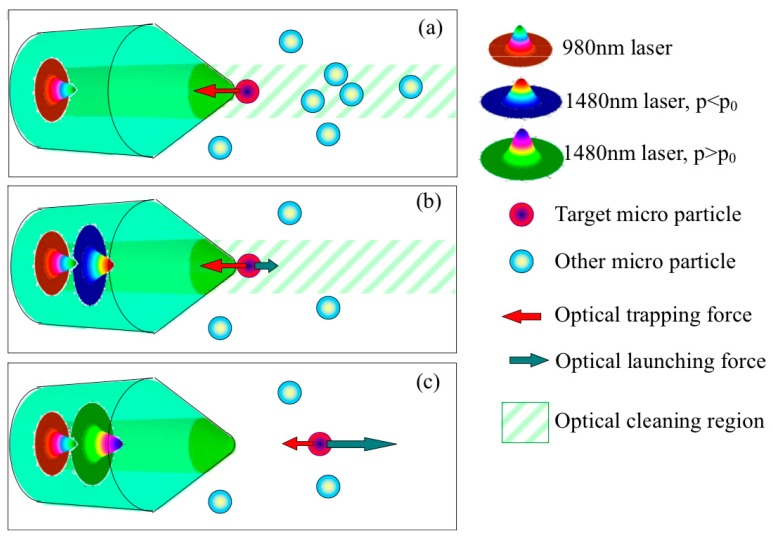
Schematic diagram of the dual-wavelength single fiber optical tweezers [39]. (**a**) Optical trapping force generated by the 980 nm laser beam. (**b**) Optical launching force generated by the 1480 nm laser beam, where the power of the 1480 nm laser beam is smaller than the power of the 980 nm laser beam, and thus the target is trapped while other objects are blown away. (**c**) If the power of the 1480 nm laser beam is larger, the launching force can launch the target away with a certain velocity.

Yuan et al. reported a series of pioneering studies and demonstrated several new methods to solve this problem. In 2013, they first demonstrated controllable SSOT without moving the fiber [37]. A yeast cell can be manipulated for a distance of approximately 3 μm with the power ratio of a fundamental mode beam (LP01) and the low-order mode beam (LP11) generated in a normal single-core fiber taper. Then, they achieved multidimensional manipulation by using the LP11 mode beam excited with a special fiber taper [38]. In 2015, they achieved optical trapping and launching based on dual-wavelength single fiber optical tweezers [39]. As shown in Figure 3, a 980 nm laser was used to trap the object towards the fiber taper, and a 1480 nm laser was used to launch the object with a certain velocity. The trapping force and the launching force can be controlled independently with different laser powers at different wavelengths.

An SSOT based on microfibers or nanofibers can achieve long-range manipulation due to its surface evanescent fields. Li and his coworker have done much research in this field and produced many theoretical and experimental results [40,41,42]. A nanofiber can trap the objects on its surface by the optical gradient force and propel trapped objects along the surface by the optical scattering force [42]. Further, as the evanescent field around the nanofiber can interact with the surrounding atoms, SSOT based on nanofibers can be used as an effective tool for atom manipulation and detection [43,44,45,46,47]. Recently, Li et al. developed a special nanofiber by embedding a silver nanowire (AgNW) into a polymethyl methacrylate (PMMA) nanofiber [40]. This nanofiber achieved trapping with a low laser power with a broad wavelength range because the AgNW enhanced the optical gradient force.

An SSOT based on a single optical force can trap a single object on or close to the fiber taper in a 3D fashion with a strongly focused beam. A manipulation length of several micrometers can be achieved with optical mode multiplexing and wavelength multiplexing, since the structure of the fiber taper is critical for functional performance, which also causes defects, such as the poor reconfigurability of devices and a short manipulation length [48,49,50].

#### 2.1.2. SSOT Based on Dual-Optical Force 

**Figure 4:**
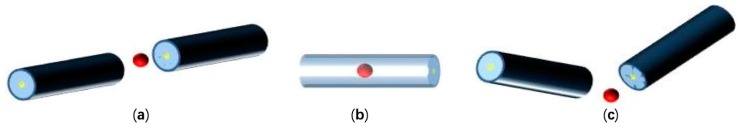
Optical trapping micro-objects based on a dual-beam fiber trap. (**a**) Aligned fibers; (**b**) Hollow-core photonic crystal fiber; (**c**) Inclined fibers.

A dual-beam fiber trap (DFT). This DFT has the special advantage of stable and flexible manipulation between two laser beams based on its fiber. The typical structures of DFTs are shown in Figure 4. As shown in Figure 4a, the primary DFT can achieve long-range optical manipulation between two aligned flat or lensed fiber tips with the help of two optical forces. The aligned fiber tips provide two coaxial and opposite optical forces for the object, and the magnitude of optical force is determined by laser power and the distance between the fiber tip and the object. In this case, the position of the object can be controlled by the balance point of the optical forces, which can be easily adjusted by the laser power from each fiber [30]. A misaligned DFT further increases the manipulation dimension to implement precise object rotation [31]. In addition to the structure based on two strictly collimated fibers, hollow-core photonic crystal fiber (HC-PCF) [32] (Figure 4b) and inclined fibers [33] (Figure 4c) can also form a dual-beam trap with more functional advantages.

**Figure 5:**
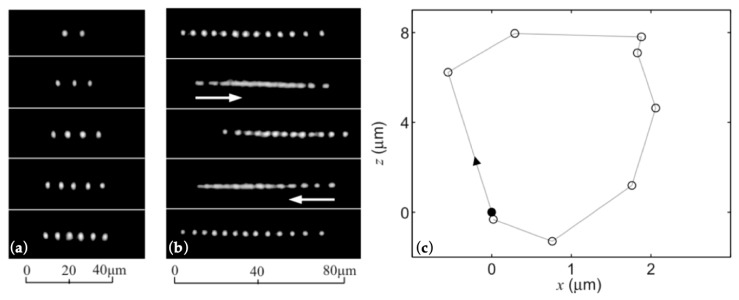
Multiple trapping and manipulation with the dual-beam fiber trap [51]. (**a**) Optical trapping of multiple objects. (**b**) Optical oscillating of a linear array of 13 objects. (**c**) Trajectory of the outermost polystyrene microsphere (PSM) for eight PSMs. The laser power of each fiber is 100 mW at 980 nm, and the objects are PSMs of with 1 μm diameters.

In [51], a DFT was used for multiple object trapping and manipulation, as shown in Figure 5. Two aligned fibers with flat facets were separated by 160 μm, and each fiber emitted 100 mW of laser with two 980 nm laser diodes, which created a platform for optical trapping and manipulation. Figure 5a shows multiple polystyrene microspheres (PSMs), with 1 μm diameters, stably trapped between two fiber ends. The PSMs form linear arrays by themselves and get closer with more PSMs. When the number of PSMs is 13 or more, they start self-sustained oscillations with a range of 20 μm for a period of 0.5 s, as shown in Figure 5b. The DFT with an offset of about 5 μm can keep PSMs oscillating as a loop. The trajectory of the outermost PSM for eight-PSM oscillation loops is shown in Figure 5c. 

In addition to optical trapping and manipulation, DFT may also deform a soft object, such as a living cell. Guck and coworkers achieved measurement of cell membrane elasticity with a unique DFT [25]. They stably trapped a single cell in a DFT formed by two SMFs. Then, they stretched it along the optical axis because the force on each side of the cell membrane may be about several hundred pN. Additionally, the unfocused laser beams can avoid thermal damage to the living cell, even if the laser power is several watts. This has potential use in biological and medical research.

DFT can also be used for sensing. Force sensing was achieved by DFT with an inclination angle (*θ*) [52]. The inclination angle (*θ*) between the two fibers was used for function selection. For object trapping, *θ* should be ≤45°, and for object lifting and force sensing, *θ* should be ≥50°. A temperature sensor was developed with a trapped microparticle in the DFT [30]. The DFT was formed by two aligned fibers with concave tips and sealed in a quartz capillary. For temperature sensing, they used a 980 nm laser to adjust the position of the microparticle and a 1550 nm laser to form the interference spectra for sensing. Li and his coworkers also demonstrated an inclined DFT with two optical fiber tapers for cell regulation and analysis [53,54]. One fiber taper was used for trapping the cell or forming cell chain. The other fiber taper was used to manipulate the targeted cell. This method has the potential for investigation of cell growth, the intercellular singling pathway, and pathogenic processes.

As fabrication technology improves, SSOTs based on special constructions, such as multi-core fibers, PCFs, and nanofibers, are proposed with better performance. Multi-core fibers can increase the manipulation dimension combined with a special fiber taper [55]. Yuan and his coworkers used a dual-core fiber [53], four-core fiber [56], and coaxial core optical fiber [57] to achieve controllable optical manipulation, oscillation, and object shooting. Cristiani et al. proposed an SSOT based on a multicore optical fiber [58]. The cores were shaped with the proper angles to reflect the laser beams into a tight focus, which is the SSOT. A strong gradient optical force generates the SSOT for 3D trapping. This SSOT can trap and manipulate microparticles over a relatively long distance with better flexibility than a DFT. The hollow-core photonic crystal fiber (HC-PCF) is an excellent carrier for optofluidics, as it is a combined channel for both laser and microfluid. A centimeter-scale long distance optical manipulation was achieved by HC-PCF [59]. A focused laser beam vertical to the fiber was used to trap the object in front of the core of the HC-PCF, and then a horizontal laser beam was used to push the object in the HC-PCF. After that, the object could be manipulated along the HC-PCF with the laser power from each end of the HC-PCF. Additionally, this research could lead to a promising new approach for biomechanical detection, because it can achieve cell deformation with the help of shear force. SSOT formed by HC-PCF can also be used for sensing. The multiple-parameter sensing of temperature, transverse mechanical vibration, and electric/magnetic fields was achieved with the help of a trapped object in the HC-PCF [32]. The object was trapped and adjusted to the sensing area with the counter-propagating laser in the HC-PCF. The change of optofluidics was reflected by a back-scattered light. 

SSOT based on a single optical force is easy to integrate and move but has the disadvantage of a short and fixed trapping/manipulation range. An SSOT based on dual-optical force uses two counter-propagating laser beams to effectively extend its trapping/manipulation range. Moreover, this method reduces the requirements of the fiber facet, thus makes the system simpler and reconfigurable for fabrication and also harmless to the trapped object. However, SSOT based on fiber inherently has an inflexible manipulation range as it uses optical force only to control its trapping position. Introducing the flow force in microfluidics is helpful to achieve a flexible and controllable scheme. To be specific, by adjusting both the optical force and flow force, the object can be manipulated along the optical axis, as discussed below.

### 2.2. Stable Combined Optical Trap with Optical Force and Microfluidic Flow Force

In this sub-section, we will introduce a stable combined optical trap (SCOT), which is formed with the combination of optical force from a single optical fiber and the flow force from the microfluid. Optofluidic applications based on SCOT will be introduced in two categories according to the type of optical force. One is a SCOT based on the optical scattering force from the fiber tip, and the other is a SCOT based on the optical gradient force from optical microfibers.

#### 2.2.1. SCOT Based on Optical Scattering Force

**Figure 6:**
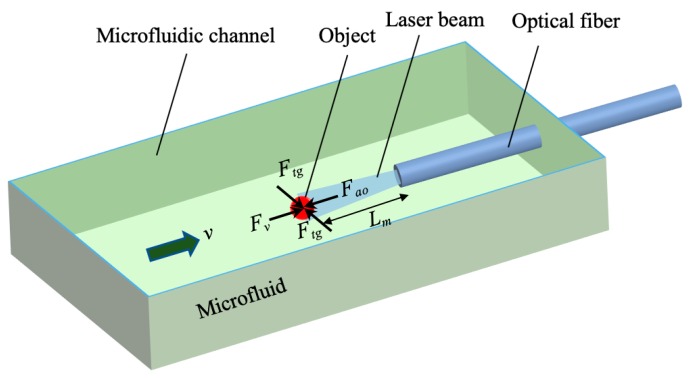
The principle of the stable combined optical trap based on a single optical fiber tip.

The principle of the SCOT near the optical scattering force is shown in Figure 6. The optical manipulation, along with the optical axis, is based on the force balance on the object between the axial optical force, *F_ao_*, and the microfluidic flow force, *F**_v_***. The flow force can be calculated by Stokes law,

*F**_v_*** = *k*_1_*v*. (1)

Here, *k*_1_ = 6π*ηa*, where *η* is the coefficient of viscosity of water, and *a* is the radius of the microparticle. As the direction of *F**_v_*** is the same with the microfluidic flow, *F**_v_*** is directed toward the fiber tip. In contrast, *F_ao_*, consisting of the scattering force, *F_as_*, and the axial gradient force, *F_ag_*, forms a counter force to push the object away from the fiber end. The *F_ag_* is negligible compared to *F_as_*, due to low acceleration of light intensity generated by the optical fiber tip. *F_ao_* is directly proportional to the laser power and inversely proportional to manipulation length, *L_m_*, which is the vertical dimension between the center of the microparticle to the fiber tip. The object can be trapped at a certain *L_m_*, corresponding to the position of the SCOT, because the total force on the object is zero. This process can be described as

*F**_v_***(*v*) = *F**_ao_***(*L_m_*) (2)

Henceforth, *L_m_* can be controlled by both the flow rate (*v*) and the laser power (*P*), and *v* can also be calibrated by *P* and *L_m_*.

In comparison to the SSOT on the fiber taper, the SCOT over the fiber tip possesses the advantage of being easy to fabricate, flexible to manipulate, and compactable to be integrated. Moreover, it greatly extended the manipulation length based on the balance between the optical force and fluid flow and shows the potential for truly 3D optical manipulation. 

The SCOT over the fiber tip can achieve controllable manipulation of single object with a long range along the optical laser beam. Gong and his coworkers made much progress in this respect. They achieved long range optical manipulation with the graded-index fiber (GIF) due to its periodic focusing effect [29]. In [60], a controllable manipulation length of over 177 µm was achieved by integrating a GIF taper with a microcavity. In this work, the manipulation length *L_m_* was directly controlled by adjusting the laser power, the flow rate, or the length of the air cavity (*L_c_*), where the air cavity was formed by the two flat fiber ends of the GIF and SMF.

**Figure 7:**
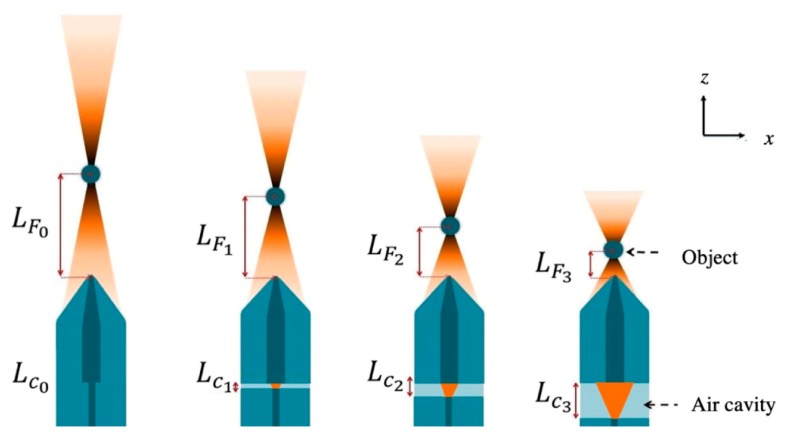
Principle of controllable optical manipulation based on air cavity length.

Figure 7 shows the principle of manipulation based on the air cavity (*L_c_*). In brief, the *L_c_* affects the incident angle and the coupling intensity of the laser beam from the SMF to GIF. Due to the periodic focusing effect of the GIF, the different incident angle and the coupling intensity produced a different light distribution from the GIF taper. The optical force was controllable, and the object could be manipulated according to the newly balanced SCOT.

**Figure 8:**
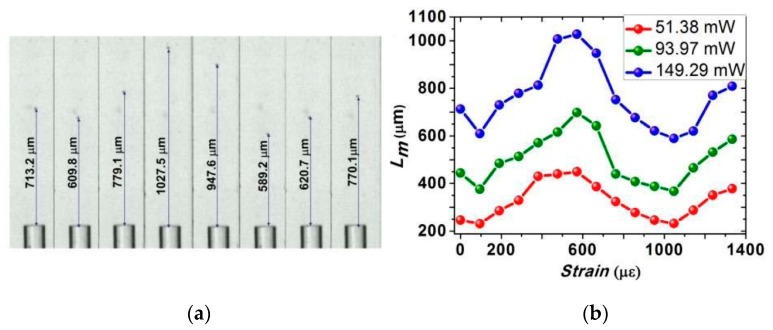
(**a**) Microscopic images of strain controllable optical manipulation by increasing the strain to 0, 95 με, 286 με, 571 με, 667 με, 1048 με, 1143 με, and 1238 με, respectively. (**b**) Manipulation length versus strain at flow rate of v = 150 nL/min [61].

In 2016, a strain controllable optical manipulation was proposed with a longer range up to 1314.1 μm [61]. This method manipulates the object by directly stretching the GIF. As the light beam converges and diverges periodically in the GIF, the change of fiber length can be used to control the distribution of the emergent field. Figure 8a shows a sequence of microscopic images of stretching the 52.5 cm GIF with a step of 50 μm. Compared to the optical manipulation of the SCOT with an optical fiber taper, the method in [61] consists of a simpler fabrication process with high repeatability and more stable performance. Figure 8b showed that the manipulation length (*L_m_*) changes by controlling the strain on the GIF.

Although the GIF provides a new optical manipulation method based on the periodic focusing effect, it is difficult to mass-produce it with consistent performance by precisely controlling the GIF length. The distribution of the emergent field is sensitive to the length of the GIF, so the performance of optical manipulation may vary substantially even with very slight difference between each GIF. 

The introduced flow force reduces the requirement of the light convergence so that the SCOT can be achieved with a flat SMF. This is the simplest scheme, with the advantage of being easy-to-fabricate and use, having high uniformity and availability for mass production, and low cost. Optofluidic flow rate detection was also achieved with a structure similar to that in Figure 6. According to Equation (2), the flow rate can be calculated by laser power or manipulation length [61,62,63].

**Figure 9:**
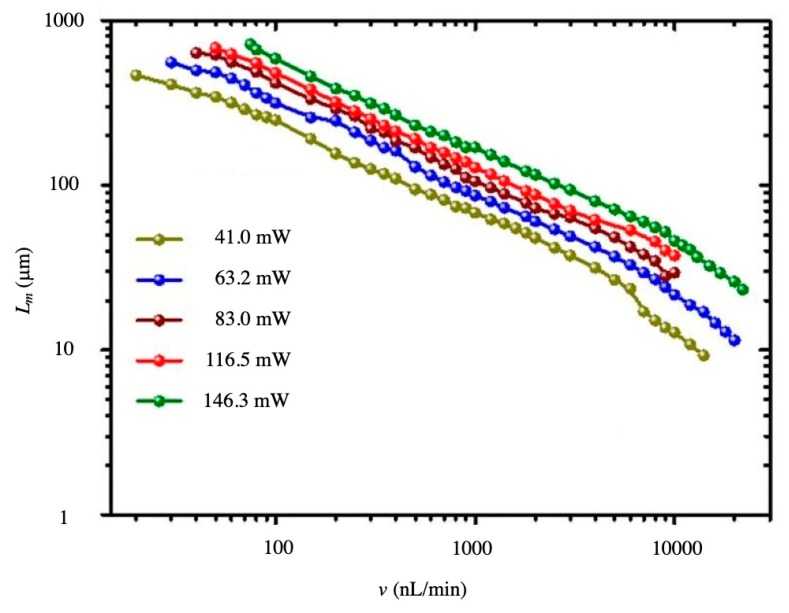
The performance of the flow rate sensing with the stable combined optical trap (SCOT) based on the cleaved single mode fiber (SMF) [62].

**Figure 10:**
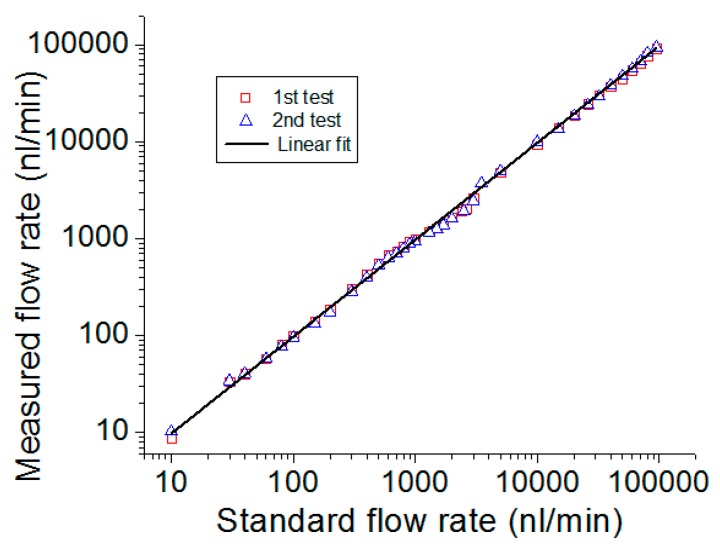
Sensing performance of the dual-mode flowmeter [63].

The performance of the flow rate sensing with SCOT based on the cleaved SMF is shown in Figure 9 [62]. By coordinating the proper laser power, this device can detect the flow rate in a large dynamic range from 20 nL/min to 22 μL/min and can manipulate the object from 3 μm to 715 μm. The method calculating the flow rate with the laser power is named the open-loop mode, which is particularly useful for detecting a low flow rate but limited for detecting a high flow rate, because the manipulation length, *L_m_*, is reversely proportional to the flow rate, *v*. In this case, the dual-mode detection induced a closed-loop mode method and enlarged the dynamic-range by four orders of magnitude, from 10 nL/min to 100,000 nL/min [63]. The sensing performance of the dual-mode flowmeter is shown in Figure 10. The mode switching threshold was set as 5000 nL/min with an initial laser power of 23.5 mW.

**Figure 11:**
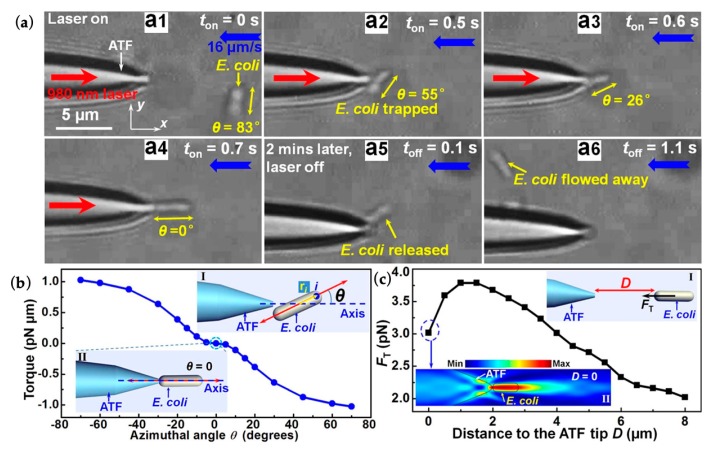
Optical trapping of a single *E. coli cell* [64]. (**a**) Optical microscope images of the trapping and orientation process of a single *E. coli* cell. The blue, red, and yellow arrows indicate flow velocity (16 mm/s), the input laser with an optical power of 30 mW, and the orientation of the *E. coli*, respectively. (**b**) The calculated torque acting on an *E. coli* cell as a function of azimuthal angle. (**c**) Calculated trapping force (*F_T_*) exerted on a single *E. coli* cell as a function of distance (*D*) between the cell and the fiber tip.

Optical trapping and orientation were achieved with an abruptly tapered SMF [64]. Microfluid delivered the *Escherichia coli* cell to the fiber tip with a velocity of 16 μm/s. A single *E*. *coli* cell was trapped with a laser power of 30 mW at a 980 nm wavelength, as shown in Figure 11a. The cell with an arbitrary azimuthal angle *θ* (Inset I of Figure 11b) was trapped with a fixed orientation in the final stable state. Figure 11b shows the calculated restoring torque on the object, with a *θ* at the central axis of the fiber tip. As shown in inset II of Figure 11b, the most stable orientation for trapping occurred at *θ* = 0, as the torque is 0. Figure 11c reflected the trapping ability of the abrupt tapered SMF. The cell was trapped at *θ* = 0 (Inset I of Figure 11c) with the energy density distribution simulated as Inset II of Figure 11c. It can be seen that the cell can be manipulated with a range of less than 8 μm and with a trapped optical force of more than 2 pN when the velocity of microfluid is 16 μm/s.

**Figure 12:**
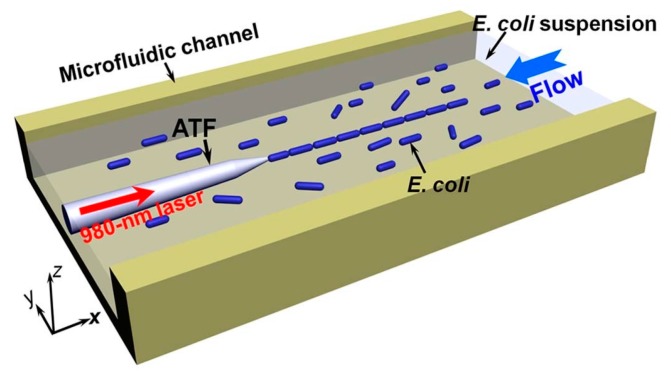
Schematic of cell–cell contact realization and retaining process. A laser at 980 nm was launched into an abrupt tapered fiber (ATF), which was placed in the microfluidic channel with a flowing suspension of *E*. *coli* cells. Multiple cells were trapped and connected in order at the tip of the ATF [64].

The SCOT over the fiber tip can also achieve the organization and transport of multiple objects. In 2013, Li and his coworkers reported an optofluidic method for realizing and retaining stable cell–cell contact and controlling the trapped cells number using an abrupt tapered fiber (ATF) [64]. As shown in Figure 12, an optical power of 30 mW at a 980 nm wavelength was launched into the ATF. Cells delivered by the microfluid with a flow velocity of 3 μm/s were trapped onto the fiber tip one after another, thereby forming a highly organized cell chain. All the trapped and connected cells were aligned with the same orientation. In 2017, they achieved controllable organization of the cell chain with a large-tapered-angle fiber probe, and demonstrated the performance with *E. coli* cells, yeast cells, and human red blood cells [65]. The cell chain can be moved by a change of laser power and flow rate.

**Figure 13:**
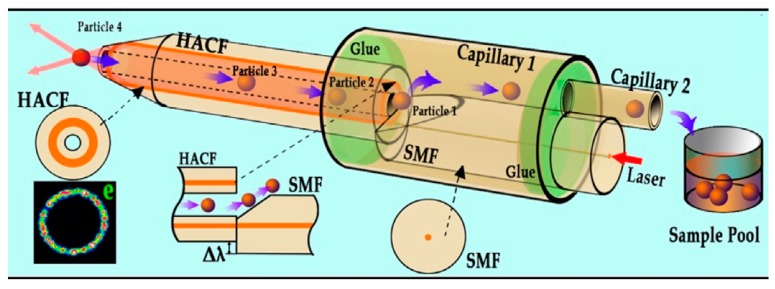
Schematic diagram of optical manipulation based on the hollow annular-core fiber taper (HACF) [66].

Microstructured optical fibers have customized structures for some unique applications. A hollow annular-core fiber taper (HAFC) was used to manipulate and transport living cells [66]. A schematic diagram of optical manipulation based on the HACF is shown in Figure 13. The hollow structure of the HACF helps to realize the sterile transport of particles in the optical fiber and provides a flow force by liquid viscous resistances (LVR). LVR is determined by the size of the object and the relative flow rate. Thus, the HACF tweezers were used in object selection and manipulation. Moreover, it is easy to clean the fiber probe and convenient for repeated use.

A SCOT based on optical scattering force can achieve controllable long-range optical manipulation and a sensitive flowmeter with an optical fiber probe. A larger optical scattering force is generated by laser irradiation from the fiber probe, which can be balanced with the flow force generated by the microfluid from the opposite direction of the laser irradiation. The optical scattering force can be adjusted by the laser power, and the flow force can be related to the flow rate. In this case, controllable long-range optical manipulation is achieved by adjusting the optical laser’s power and flow rate, and the flow rate is calculated from the manipulation length or the laser power.

#### 2.2.2. SCOT Based on Optical Gradient Force

A SCOT around the microfiber could achieve long-range manipulation along the fiber. The optical gradient force plays a crucial role in optical trapping based on SCOT. A microfiber with a subwavelength diameter enhances the evanescent field and exerts a large optical gradient force perpendicular to the fiber surface. When the microfluid flows against the optical gradient force, the object can be trapped with the combination of optical force generated by the light leaked from the optical fiber and the dragging force induced by the fluidic flow and then move along the surface via the optical scattering force that occurs in the direction of light propagation [67]. 

**Figure 14:**
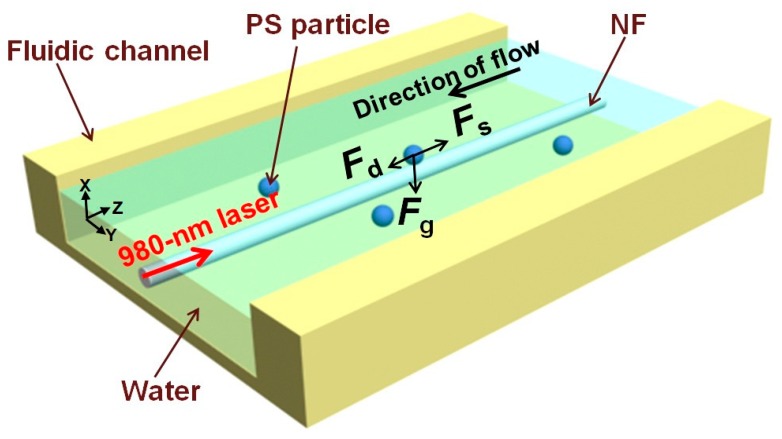
Schematic diagram of the experiment. *F_d_* shows the drag force on the particle (in blue) induced by the fluidic flow. *F_g_* and *F_s_* denote the gradient and scattering forces, respectively, from the evanescent field [68].

A SCOT around the microfiber can achieve optical transport along the fiber with the help of flow force, optical gradient force, and optical scattering force. Li et al. reported the backward optical transport of Polystyrene (PS) nanoparticles (713 nm in diameter) using an optical nanofiber with a diameter of 710 nm [68]. Figure 14 shows the schematic of the experiment. The optical forces, including gradient force (*F_g_*) and scattering force (*F*_s_), were generated and controlled using a diode laser with a 980 nm wavelength, and the flow force (*F_d_*) induced by the microfluid was dependent on flow rate. When the laser was on, the evanescent field of the nanofiber applied *F_g_* and *F*_s_ to the PS particle. *F_g_*, directed towards the stronger optical intensity region, traps the particles to the surface of the nanofiber. The *F*_s_ with a direction parallel to the light propagation propels the particle to move along the nanofiber. By varying the laser power from 0 to 90 mW and flow velocity from 0 to −20 μm/s, the backward transport velocity exhibits a linear dependence. Furtherly, bidirectional optical transport can be achieved with two counter-propagating laser beams from each end of the optical nanofiber [42]. The transportation direction and velocity of the particles can be controlled by changing the difference between the laser power from each side of the nanofiber.

**Figure 15:**
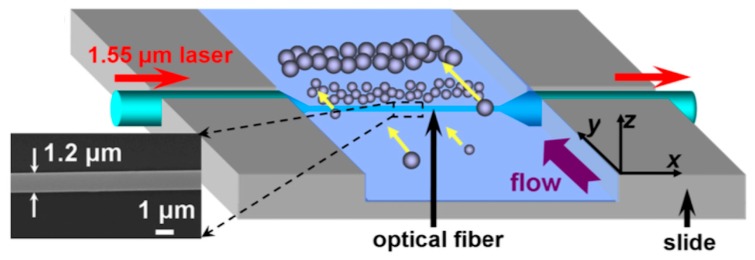
Schematic diagram for particle separation in fluidic flow by an optical fiber. The inset shows the scanning electron microscope image of a 1.2 μm optical fiber [69].

Following this scheme, a particle separation method was demonstrated [69]. Figure 15 shows a schematic of the SCOT around the microfiber. A 1.55 μm laser was launched into the microfiber for particle separation. The microfiber with a 1.2 μm diameter was placed in a channel. The suspensions flowed into the channel for separation. Three types of particle mixtures were successfully separated, including 5/10 μm PMMA particles, 2.08 /5.65 μm SiO_2_ particles, and 2.08 μm SiO_2_/yeast cells.

In conclusion, a SCOT based on optical scattering force (*F_s_*) achieves force balance within a small cross-section along the optical axis. Thus, it can accurately trap or manipulate a single object, even far from the fiber probe. A SCOT based on optical gradient force (*F_g_*) achieves force balance vertical to the microfiber surface. Thus, it can simultaneously trap or manipulate a larger number of objects. 

The major difference between a SCOT and SSOT is that a SCOT utilizes the flow force in the microfluidic system as an extra control factor. By tuning the flow force, a SCOT can achieve more flexible and longer-range trapping or manipulation, without requiring sophisticated fiber structures as a SSOT does. In addition, a SCOT can calibrate the flow rate by measuring the laser power and the manipulation length. 

## 3. FOF Technology Based on a Photothermal Effect

Photothermal effects can be used for optical manipulation and sensing in optofluidics. The photothermal effect is usually weak and needs to be enhanced by increasing laser radiation or absorption. In FOFs, laser radiation is usually increased by applying a microfiber, and laser absorption is usually increased by integrating special materials with different components of the optofluidic system. 

### 3.1. FOF Technology Based on Photothermal Effect with Microfibers 

In a standard optical fiber, the optical field is well confined to the fiber core, unable to interact with microfluid from the side [70]. Therefore, the key to generating a photothermal effect is improving the radiation of the optical laser from the fiber core to the microfluid. The optical microfiber provides an efficient solution for radiation enhancement, which has been extensively investigated.

Optical microfibers can easily be fabricated from commercial optical fibers by heating at a melting temperature and stretching to an appropriate size, enabling much higher flexibility and compatibility over conventional fiber-based systems [71,72]. Laser at a wavelength with high absorption to the solution is often launched into the microfiber to further enhance the photothermal effect. This can achieve optical manipulation and sensing for optofluidic applications. 

The photothermal effect can achieve massive particle trapping and manipulation based on its derivative effects, i.e., the photophoresis effect and temperature gradient effect. The photophoresis effect is generated by an uneven heat distribution when the photothermal effect acts on the particles in the microfluid [73]. Uneven heat distribution will increase the movement of the surrounding water molecules, and eventually generate negative photophoresis or positive photophoresis to drive the particle towards or away from the light source, separately. The temperature gradient effect is based on the strong laser absorption of the liquid in the microfluid and can drive the particles to move to the colder region [74]. 

**Figure 16:**
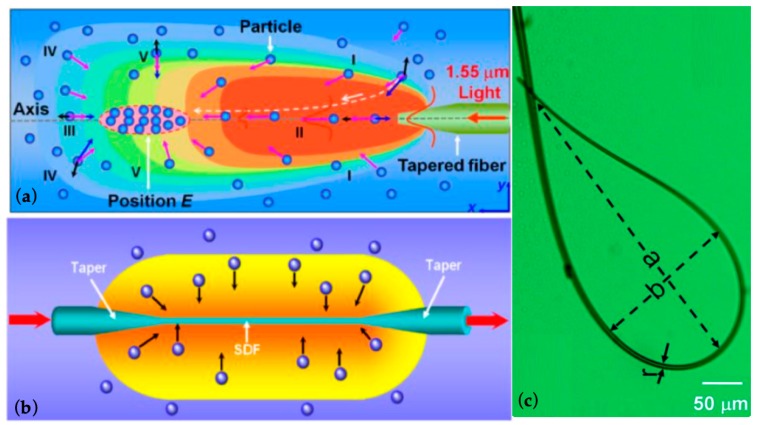
Massive photothermal trapping and assembly of particles using (**a**) tapered optical fiber [75], (**b**) subwavelength diameter optical fiber [76], and (**c**) optical fiber ring [77].

Li and his co-workers have been achieved massive photothermal trapping and manipulation with different structures, such as tapered optical fiber (TF) [75], subwavelength diameter optical fiber (SDF) [76], and optical fiber ring (FR) [77], as shown in Figure 16 Using a TF with a diameter of 3.1 μm for the taper (Figure 16a), plenty of particles were assembled into a spindle-shaped region, when the laser power was 170 mW at 1550 nm. There was a space of about 380 μm between the assembled particles and the TF after 15 min. Using an SDF (Figure 16b), particles were assembled around the SDF with a laser power of 200 mW, and reached saturation after 360 s. With an FR (Figure 16c), particles were trapped and assembled in the center of the FR with a power of 97 mW. 

Photothermal effect can achieve optofluidic sensing based on the evanescent field around the microfiber during laser transmission. The microfiber with a diameter of several micrometers or less can enhance the evanescent field and is sensitive to the ambient temperature around it [78].

**Figure 17:**
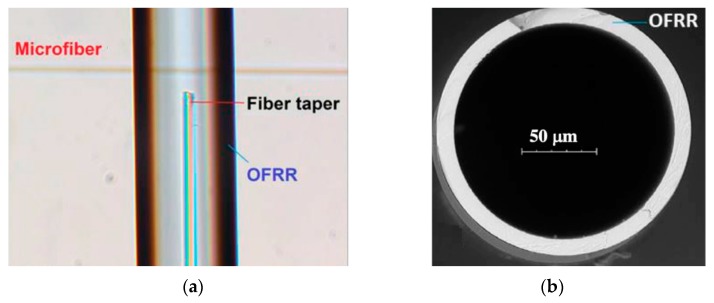
(**a**) The side view and (**b**) the cross section of the sensor of the optofluidic ring resonator [79].

An optofluidic flow rate sensor based on the photothermal effect in a microfluid has been proposed by Gong and coworkers [79]. The side view and the cross section of the sensor are shown in Figure 17. A microfiber with a waist of approximately 3 µm was fabricated by heating and drawing a commercial SMF. A hollow round capillary acted as an optofluidic ring resonator perpendicular to the microfiber. A small fraction of the incident light of the microfiber was coupled into the capillary due to the evanescent field and kept circulating in the wall due to the total reflection of the smooth inside of the round capillary. A part of the reflection was coupled into microfiber and transmitted to the detector. The wavelength shift of the transmission spectrum can be used as a function of the flow rate. As the full width at half magnitude (FWHM) of the linewidth is narrow, this structure can achieve flow rate sensing with high sensitivity. 

A laser at 1480 nm coupled into the fiber taper was used to heat the liquid in the capillary for temperature change. The fiber taper can be easily fabricated with a commercial fiber by different methods, such as chemical etching, mechanical polishing, and flame heating. Since the fundamental mode of the evanescent field was powerful due to its larger radius near the capillary and the effective index (*n_eff_*), it has often been chosen as the output for sensing. The microfiber was close to the outside of the round capillary. Therefore, while the fundamental mode can act on the capillary, it cannot pass through to change the parameters of the microfluid. As a result, the difference of temperature can be calibrated with the relative wavelength shift as

(3) $$\frac{\Delta \lambda }{\lambda }=\left(\alpha +\frac{\kappa_{wall}}{n_{eff}}\frac{\partial n_{eff}}{\partial n_{wall}}\right)\Delta T$$

where *α* is the thermal expansion coefficient of the resonator, which can be calculated by 1/*r* (∂*r*/∂*T*), and $\kappa_{wall}=\partial n/\partial T$ is the photothermal effect coefficient of the capillary. The photothermal effect occurs near the fiber taper. First, the temperature of the microfluid increased near the fiber taper and then transferred to the round capillary. The wavelength shift is dependent on two factors in Equation (3): the thermal expansion of the capillary and its photothermal effects. 

Optical microfibers can enhance the effective photothermal effect for manipulation and sensing applications. For optical manipulation, the photothermal effect enables massive objects manipulation with high flexibility. For optical sensing, the photothermal effect could be employed together with another microresonator for local detection of the microfluidic flow rate with high sensitivity by detecting the wavelength shift.

### 3.2. FOF Technology Based on a Photothermal Effect with Special Materials

Recently, materials with strong laser absorption, such as gold nanoparticles (Au NPs), graphene oxide (GO), and carbon nanotubes (CNTs), have been extensively investigated to improve the efficiency of photothermal conversion. These kinds of materials can enhance the photothermal effect for FOF to achieve applications of optical manipulation and sensing [80]. Photothermal materials are mostly integrated with optical fibers or microfluids, as introduced below.

#### 3.2.1. Materials Integrated with Optical Fiber (MIFs)

It is difficult to generate an optimal photothermal effect with an untreated optical fiber due to its low light-thermal conversion efficiency. Integrating photothermal materials with the fiber (MIF) is a useful method to improve the photothermal effect. MIF can achieve optical trapping and manipulation based on the photothermal materials coated on the optical fiber tip or on the cylindrical surface. 

**Figure 18:**
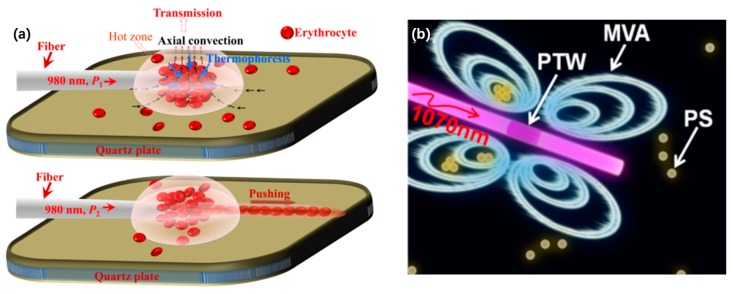
Schematic diagrams of optical trapping and manipulation based on the photothermal materials coated (**a**) on the optical fiber tip [81] or (**b**) on the cylindrical surface [82].

Xing and co-workers investigated an optical manipulation method based on a graphene-coated microfiber probe (GCMP) [81]. As shown in Figure 18a, a 980 nm laser coupled into the GCMP can effectively trap erythrocytes based on photothermal effect induced thermophoresis and natural convection flow and can arrange the trapped erythrocytes over a long distance, combining with the optical scattering force. The MIF of the graphene oxide on the cylindrical surface of the fiber achieved mobile vortex arrays with high stability for the no-time-delay, non-contact delivery of massive trapped objects along the arbitrary direction [82], as shown in Figure 18b. When a 1070 nm laser was coupled into the coated fiber, a temperature gradient was generated and excited the oscillatory wave to trap and deliver the particles. 

Optical sensing can also be achieved with MIF. Fiber optofluidic microbubble-on-tip (μBoT) sensors, featuring a flat fiber tip coated with carbon nanotube (CNT) film [83] or gold nanofilm [84], have been proposed. The process and sensing mechanism are mainly based on a reconfigurable microbubble. When the laser was irradiated on the coated fiber tip, a microbubble was generated and gradually expanded. The generation of the microbubble can be monitored using both the microscope and interference spectrum of the interferometer formed by the fiber tip and the surface of the microbubble, and the changes of parameters in microfluidics can be calibrated with the growth rate of the microbubble. 

A CNT-coated μBoT sensor can detect the temperature and flow rate with the laser at 980 nm. The sensing signal was the free spectral range (FSR) of the microbubble heated with the same duration. As the diameter of the µBoT interferometer increases over time, the FSR decreases. For temperature sensing, the microbubble expands mainly based on gas generation from liquid vaporization around the fiber tip. The principle of flow rate sensing is mainly based on microbubble expansion with the dissolving gas in the flow fluid.

**Figure 19:**
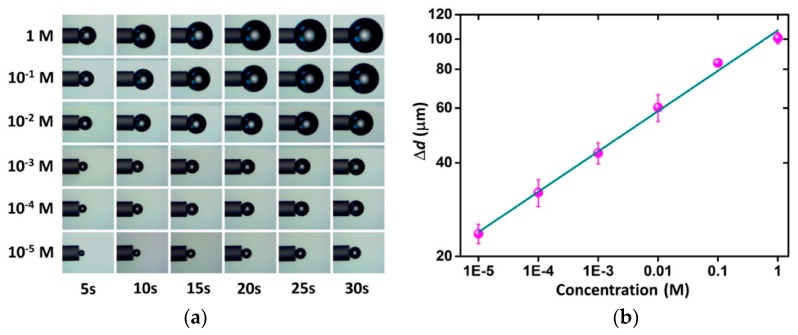
(**a**) Microscopic images of the microbubbles and (**b**) H_2_O_2_ concentration detection [84].

A gold-coated μBoT sensor can detect the concentration of the solution with a laser at 1550 nm. Sucrose and H_2_O_2_ were chosen as models to demonstrate sensing performance, which represents two different sensing mechanisms. One is based on the evaporation of liquid near the fiber tip, and the other is based on heat-induced chemical decomposition. For sucrose sensing, the μBoT sensor achieved a dynamic range of two orders of magnitude, from 0.5 wt% to 50.0 wt%. For H_2_O_2_ sensing, the μBoT sensor achieved a dynamic range of five orders of magnitude, from 10^−5^ M to 1 M, as shown in Figure 19. The microscopic images of the microbubbles generated with different concentrations of H_2_O_2_ were recorded at different heating times (Figure 19a). The imaging method was chosen due to its low cost, and ∆*d* as a function is shown in the log-log scale in Figure 19b.

The result of sensing shows a large dynamic range and high sensitivity, which demonstrate the high performance of sensing based on this mechanism. This is the first report on concentration sensing based on a reconfigurable µBoT structure. This technique shows many advantages for optofluidic detection, such as flexibility, reconfigurability, low cost, ease of fabrication, and ease of use.

Besides coating the photothermal materials on the fiber tip, compact fiber-optic sensors also can be achieved by integrating materials into the optical fibers. A miniature, all-optical, fiber-optic sensor has been demonstrated for thermal conductivity measurements [85]. A vanadium doped fiber spliced with an SMF was used as a highly absorbent part and coated with a thin zirconia film to create a semi-reflective surface. Thus, the short section of vanadium doped fiber formed an all-fiber F–P interferometer for sensing. The all-silica design makes the sensor compatible with most chemical environments and has good potential for use at elevated temperatures and high pressures.

#### 3.2.2. Materials Integrated with Microfluids

Materials with strong laser absorption abilities can also be directly integrated with microfluids, such as the channel of microfluidics or the objects in the microfluid. This method increases photothermal conversion efficiency and shortens the response time for manipulation or sensing.

**Figure 20:**
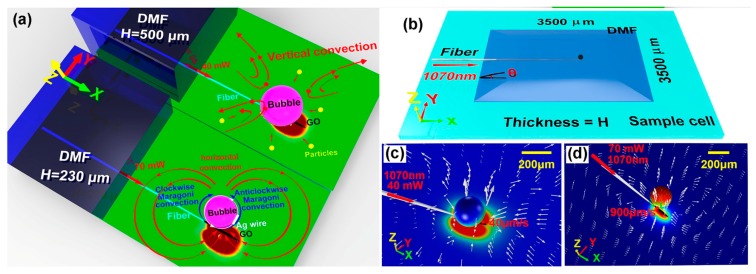
Simulation of thermal convection when the bubble is located at H = 230 μm and 500 μm and P = 40 mW and 70 mW [93]. (**a**) Schematic model of different functional flows. (**b**) Simulated model of the microfluidic system. (**c**) Vertical convection when a 160 μm diameter microbubble is located above the heater with T = 400 K at H = 500 μm and P = 40 mW. The longest arrow shows the maximum velocity of 40 μm/s. (**d**) Horizontal convection induced by the heater when the 160 μm diameter microbubble is located at the side of the graphene oxide (GO) heater with T = 450K at H = 230 μm and P = 70 mW. The longest arrow shows that the maximum velocity of 900 μm/s. Marangoni convection (green arrows) at the surface of the bubble would influence movement of particles when the thickness of the N, N-Dimethylformamide (DMF) was less than 300 μm. Flow-induced deformation of the bubble is not considered in these simulations.

Photothermal materials on the channel have been widely applied to handling liquids [86,87,88], manipulation in microfluid [89,90,91], and micromaching processes [92] because the photothermal effect on the channel can cause fluid dynamics, phase changing, interfacial action, and a strong vertical temperature gradient. A laser induced microbubble-based device was introduced as an example of a photothermal effect on the channel [93]. Graphene oxide (GO) was integrated with the channel, which could serve as a miniature heat source to generate a microbubble and control dynamic behaviors of flow by adjusting optical laser power at the micrometer scale. A microfiber was used to simulate the photothermal effect at the locality of the microfluid with a 1070 nm CW laser. This device can be used for optical manipulation based on the thermal convection around a microbubble, which can be controlled by an optical laser. A simulation of thermal convection is shown in Figure 20. Based on controllable thermal convection, i.e., vertical convection and Marangoni convection, the microfluidic flow around the microbubble can be controlled easily, and the massive objects around the microbubble can be manipulated. 

Photothermal materials integrated with the object have received great attention because the materials can efficiently convert adsorbed photons into thermal energy, and this function can directly act on the target with minimally invasive effects [94,95]. Based on these characteristics, photothermal therapy and other bio-medical applications based on IT have been increasingly and widely investigated as facile oncological treatment methods [96]. In 2003, Halas and co-workers first demonstrated the selective destruction of breast carcinoma cells based on the target region integrated with gold-on-silica nanoshells [97]. After that, a variety of hybrid materials with different compositions or structures have been explored for photothermal therapy (PTT) [98,99]. Optical fiber used to induce laser power to a target are minimally invasive due to their flexibility, optical conductivity, and biocompatibility. 

**Figure 21:**
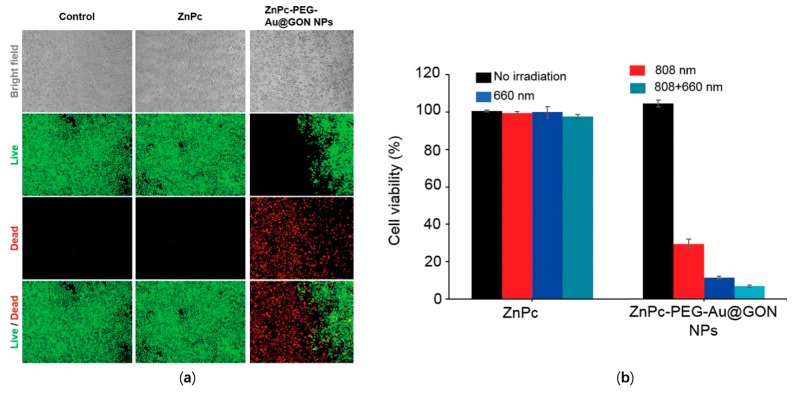
(**a**) Bright-field and fluorescence microscopy images of control, ZnPc, and ZnPc-PEG-Au@GON nanoparticle (NP) treated HeLa cells wgucg were exposed to a 660 nm fiber coupled laser with 67 mW/cm^2^ for 15 min and subsequently stained by a lince/Dead assay reagent, green: live cells, red: dead cells. (**b**) Cell viability of HeLa cells treated with ZnPc, and ZnPc-PEG-Au@GON NPs and subsequent exposure to 808 nm with 0.67 W/cm^2^ for 20 min, 660 nm with 0.2 W/cm^2^ for 10 min, or both light sources with the same condition, sequentially [98].

A hybrid material with components of zinc phthalocyanine (ZnPc), polyethylene glycol (PEG), core–shell nanoparticles (NPs), a gold (Au) core, and graphene oxide nanocolloid (GON) (ZnPc-PEG-Au@GON NPs), were successfully applied to the in vitro photothermal ablation of HeLa (Human cervical cancer cell line) cells [98]. A 660 nm fiber coupled laser was used to generate a effect on treated HeLa cells, and induced death of nearly all of them, as shown in Figure 21a. Figure 21b shows the contrast of the cell viability of HeLa cells treated with ZnPc and ZnPc-PEG-Au@GON NPs. The results clearly indicated that ZnPc-PEG-Au@GON NPs could enhance photothermal efficiency for medical treatment.

In conclusion, FOF technology based on a photothermal effect can achieve massive object manipulation and microfluid sensing with microfibers or with special materials, which enhance laser radiation or absorption, respectively. However, because light energy needs a relatively long time to be converted to heat energy, FOF technology based on a photothermal effect usually has a long response time for manipulation or sensing. Therefore, photothermal materials are often used to further increase photothermal conversion efficiency. However, this method maybe unstable in some cases, as photothermal materials could be washed away by the microfluid. 

## 4. Conclusions

This review mainly introduces the fiber optofluidic technology (FOF) based on two major opto-physical effects: optical force and the photothermal effect. Optical force is used for optofluidic manipulation and sensing with a stable single optical trap (SSOT) and a stable combined optical trap (SCOT), and the photothermal effect is used for various microfluidic control applications with an optical microfiber and special absorption materials.

SSOT and SCOT exploit different types of forces in optofluidic systems. SSOT is formed only by optical force and can be further subdivided into those based on a single optical force and those based on a dual-optical force. The former often uses fiber taper to generate a large optical force through a strong convergent laser beam, while the latter uses two beams to generate a pair of balanced optical force via two counter-propagating laser beams. SCOT is formed with a balance of optical force and flow force in the microfluid. Furthermore, according to the components of optical force balance with flow force, SCOTs can be subdivided into those based on optical scattering force (*F_s_*) and those based on optical gradient force (*F_g_*). The SCOT based on *F_s_* is often generated by a fiber tip, whose end is against the fluid flow direction. This SCOT can consciously adjust the position of the trapped object along the optical axis with a large manipulation range. Further, it can also be used for flow rate sensing with excellent performance in dynamic range and sensitivity. The SCOT based on *F_g_* is often generated by an optical microfiber, which is perpendicular to the fluid flow. This SCOT is an effective method for massive trapping and manipulation of objects at the micro/nano scale. 

The photothermal effect is used for FOF technology in two major ways: by using the optical microfiber and by using special absorption materials. The optical microfiber is often used for enhancing laser radiation and can achieve massive object manipulation, as well as for flow rate sensing. Integration with photothermal materials, such as carbon nanotubes (CNTs), and gold (Au), is a common and effective method to enhance laser absorption. Photothermal materials can be flexibly integrated with any FOF components when required. FOF technology can be integrated with fibers and generate a laser-controlled thermal field to achieve optical manipulation and sensing. Materials integrated with a channel can be used for handling liquids, manipulation in microfluid, micromachining processes, and other applications of microfluidic control. Materials integrated with a target can directly act on the target with a certain position and less thermal energy loss. This method has broad prospects in biological research and medical treatment. 

The main results of fiber optofluidic technology based on optical force and photothermal effects are summarized in Table 2 and Table 3, respectively. Using optical force and photothermal effects, FOF technology present various advantages, including easy fabrication, miniaturization, low cost, high sensitivity, and a large dynamic range. We believe that many new applications will be explored for physical, chemical, and biological use based on FOF technology in the near future [100,101]. 

## Figures and Tables

**Table 1:** A summary of sensing application based on fiber-optic interferometer.

Device Types	Structures	Applications	Ref.
FPI	Extrinsic/intrinsic cavity	RI sensing	[7]
		Temperature and humidity sensor	[8]
MZI/MI	Waist-enlarged fiber taper	Label-free biosensing	[9]
		Temperature-immune humidity sensing	[10,11]
		Temperature-immune RI sensing	[12]
MZI/MI	Core-offset	Temperature-immune RI sensing	[13]
MZI	Two micro-cavities	RI-immune temperature sensing	[14]
SI	Fiber loop mirror	Temperature-immune pressure sensing, High-temperature sensing	[15,16]

FPI is Fabry-Perot interferometer, MZI is Mach-Zehnder interferometer, MI is Michelson interferometer, SI is Sagnac interferometer, and RI is refractive index.

**Table 2:** A summary of fiber optofluidic technology based on optical force.

Device Types	Principle	Fabrication	Features
Stable Single Optical Trap based on single optical force	Strongly focused beam	Fiber taper	Single object trapping with short range [36,37,38,39]
	Surface evanescent fields	Micro/nanofiber	Massive object trapping on the fiber surface [40,41,42,43,44,45,46,47]
Stable Single Optical Trap based on dual optical force	Balance of two optical forces	Two aligned fiber probes	Object manipulation (~200 μm) [30]. Object deformation [25]
		Two misaligned fiber probes	Object rotation [31] Object oscillating, and moving around [51] Object lifting, and force sensing [52] Cell regulation and analysis [53,54]
		HC–PCF	Multiple parameter sensing [32] A centimeter-scale long distance optical manipulation, cell deformation [59]
Stable Combined Optical Trap based on optical scattering force	*F_s_* = *F_v_*	Fiber probe	Long range manipulation and large dynamic range flowmeter [60,61,62,63] Multiple object organization [64,65] Manipulation and transportation [66]
Stable Combined Optical Trap based on optical gradient force	*F_g_* = *F_v_*	Microfiber	Massive trapping, manipulation, and selection [42,67,68,69]

**Table 3:** A summary of fiber optofluidic technology based on a photothermal effect.

Device Types	Principle	Fabrication	Features
FOF technology based on a photothermal effect with a microfiber	Enhancing the laser radiation	Fiber taper	Massive particle trapping and manipulation [75]
		Micro/nanofiber	Massive object trapping on fiber surface [76] Flow rate sensing [80]
		optical fiber ring	Massive object trapping in center of ring [77]
FOF technology based on a photothermal effect with special materials	Enhancing the laser absorption	Materials integrated with the fiber	Massive object trapping and delivering [81,82] Multiple parameter sensing [83,84,85]
		Materials integrated with the channel	Microfluid control [86,87,88], massive object manipulation [89,90,91], micromaching processes [92]
		Materials integrated with the channel	Medical treatment with minimally invasive effects [94,95,96,97,98,99]

## References

[1] Schmidt H., Hawkins A.R. (2011). The photonic integration of non-solid media using optofluidics. Nat. Photonics.

[2] Fan X., White I.M. (2011). Optofluidic microsystems for chemical and biological analysis. Nat. Photonics.

[3] Sudirman A., Margulis W. (2014). All-Fiber optofluidic component to combine light and fluid. IEEE Photonics Technol. Lett..

[4] Erickson D., Sinton D., Psaltis D. (2011). Optofluidics for energy applications. Nat. Photonics.

[5] Cubillas A.M., Unterkofler S.T., Euser G., Etzold B.J., Jones A.C., Sadler P.J., Wasserscheid P., Russell P.S.J. (2013). Photonic crystal fibres for chemical sensing and photochemistry. Chem. Soc. Rev..

[6] Zhu T., Wu D., Liu M., Duan D.W. (2012). In-line fiber optic interferometric sensors in single-mode fibers. Sensors.

[7] Zhang Q., Hao P., Tian X. (2017). High-visibility in-line fiber-optic optofluidic Fabry–Pérot cavity. Appl. Phys. Lett..

[8] Liu S., Ji Y., Yang J. (2018). Nafion film temperature/humidity sensing based on optical fiber Fabry-Perot interference. Sens. Actuators A.

[9] Chen L.H., Chan C.C., Ni K., Hu P.B., Li T., Wong W.C., Poh C.L. (2013). Label-free fiber-optic interferometric immunosensors based on waist-enlarged fusion taper. Sens. Actuators B.

[10] Shao M., Qiao X., Fu H. (2014). A Mach–Zehnder interferometric humidity sensor based on waist-enlarged tapers. Opt. Laser Eng..

[11] Hu P., Dong X., Ni K. (2014). Sensitivity-enhanced Michelson interferometric humidity sensor with waist-enlarged fiber bitaper. Sens. Actuators B.

[12] Zhang S., Zhang W., Geng P., Gao S. (2013). Fiber Mach-Zehnder interferometer based on concatenated down-and up-tapers for refractive index sensing applications. Opt. Commun..

[13] Tian Z., Yam S.S., Loock H.P. (2008). Single-mode fiber refractive index sensor based on core-offset attenuators. IEEE Photonics Technol. Lett..

[14] Jiang L., Yang J., Wang S., Li B., Wang M. (2011). Fiber Mach-Zehnder interferometer based on micro cavities for high-temperature sensing with high sensitivity. Opt. Lett..

[15] Fu H.Y., Tam H.Y., Shao L.Y., Dong X., Wai P.K.A., Lu C., Khijwania S.K. (2008). Pressure sensor realized with polarization-maintaining photonic crystal fiber-based Sagnac interferometer. Appl. Opt..

[16] Moon D.S., Kim B.H., Lin A., Sun G., Han T.G., Han W.T., Chung Y. (2007). The temperature sensitivity of Sagnac loop interferometer based on polarization maintaining side-hole fiber. Opt. Express.

[17] Bertucci A., Manicardi A., Candiani A., Giannetti S., Cucinotta A., Spoto G., Konstantaki M., Pissadakis S., Selleri S., Corradini R. (2015). Detection of unamplified genomic DNA by a PNA-based microstructured optical fiber (MOF) Bragg-grating optofluidic system. Biosens. Bioelectron..

[18] Zhang N., Humbert G., Wu Z., Li K., Shum P.P., Zhang N.M.Y., Wei L. (2016). In-line optofluidic refractive index sensing in a side-channel photonic crystal fiber. Opt. Express.

[19] Zhang N., Li K., Cui Y. (2018). Ultra-sensitive chemical and biological analysis via specialty fibers with built-in microstructured optofluidic channels. Lab. Chip..

[20] Rifat A.A., Ahmed R., Yetisen A.K. (2017). Photonic crystal fiber based plasmonic sensors. Sens. Actuators B.

[21] Xing Z., Zheng Y., Yan Z. (2019). High-sensitivity humidity sensing of microfiber coated with three-dimensional graphene network. Sens. Actuators B.

[22] Vaiano P., Carotenuto B., Pisco M., Ricciardi A., Quero G., Consales M., Cusano A. (2016). Lab on Fiber Technology for biological sensing applications. Laser Photonics Rev..

[23] Ashkin A. (1970). Acceleration and trapping of particles by radiation pressure. Phys. Rev. Lett..

[24] Ashkin A., Dziedzic J.M., Bjorkholm J., Chu S. (1986). Observation of a single-beam gradient force optical trap for dielectric particles. Opt. Lett..

[25] Guck J., Ananthakrishnan R., Moon T., Cunningham C., Käs J. (2000). Optical deformability of soft biological dielectrics. Phys. Rev. Lett..

[26] Jess P., Garcés-Chávez V., Smith D., Mazilu M., Paterson L., Riches A., Herrington C., Sibbett W., Dholakia K. (2006). Dual beam fibre trap for Raman microspectroscopy of single cells. Opt. Express.

[27] Xu X., Cheng C., Xin H., Lei H., Li B. (2015). Controllable orientation of single silver nanowire using two fiber probes. Sci. Rep..

[28] Taguchi K., Atsuta K., Nakata T., Ikeda R. (2000). Levitation of a microscopic object using plural optical fibers. Opt. Commun..

[29] Gong Y., Huang W., Liu Q.F., Wu Y., Rao Y., Peng G.D., Lang J., Zhang K. (2014). Graded-index optical fiber tweezers with long manipulation length. Opt. Express.

[30] Zhang Y., Liang P., Liu Z., Lei J., Yang J., Yuan L. (2014). A novel temperature sensor based on optical trapping technology. J. Lightwave Technol..

[31] Xiao G., Yang K., Luo H., Chen X., Xiong W. (2016). Orbital rotation of trapped particle in a transversely misaligned dual-fiber optical trap. IEEE Photonics J..

[32] Bykov D.S., Schmidt O.A., Euser T.G., Russell P.S.J. (2015). Flying particle sensors in hollow-core photonic crystal fibre. Nat. Photonics.

[33] Bell A.G. (1880). On the production and reproduction of sound by light. Am. J. Sci..

[34] Terazima M., Hirota N., Braslavsky S.E., Mandelis A., Bialkowski S.E., Diebold G.J., Miller R., Fournier D., Palmer R.A., Tam A. (2004). Quantities, terminology, and symbols in photothermal and related spectroscopies (IUPAC Recommendations 2004). Pure Appl. Chem..

[35] Ashkin A. (1992). Forces of a single-beam gradient laser trap on a dielectric sphere in the ray optics regime. Biophys. J..

[36] Liu Z., Guo C., Yang J., Yuan L. (2006). Tapered fiber optical tweezers for microscopic particle trapping: Fabrication and application. Opt. Express.

[37] Liu Z., Wang L., Liang P., Yuan L. (2013). Mode division multiplexing technology for single-fiber optical trapping axial-position adjustment. Opt. Lett..

[38] Zhang Y., Liang P., Lei J. (2014). Multi-dimensional manipulation of yeast cells using a LP11 mode beam. J. Lightwave Technol..

[39] Liu Z., Liang P., Zhang Y., Zhang Y., Zhao E., Yang J., Yuan L. (2015). Micro particle launcher/cleaner based on optical trapping technology. Opt. Express.

[40] Cheng C., Xu X., Lei H., Li B. (2016). Plasmon-assisted trapping of nanoparticles using a silver-nanowire-embedded PMMA nanofiber. Sci. Rep..

[41] Li Y., Xin H., Xu X., Liu X., Li B. (2018). Fibre-optic trapping and manipulation at the nanoscale. Adv. Mater. Lett..

[42] Lei H., Xu C., Zhang Y., Li B. (2012). Bidirectional optical transportation and controllable positioning of nanoparticles using an optical nanofiber. Nanoscale.

[43] Brambilla G., Murugan G., Wilkinson J., Richardson D. (2007). Optical manipulation of microspheres along a subwavelength optical wire. Opt. Lett..

[44] Xu L., Li Y., Li B. (2012). Size-dependent trapping and delivery of submicro-spheres using a submicrofibre. New J. Phys..

[45] Zhang Y., Lei H., Li B. (2013). Refractive-Index-Based Sorting of Colloidal Particles Using a Subwavelength Optical Fiber in a Static Fluid. Appl. Phys. Express.

[46] Zhang Y., Li B. (2013). Particle sorting using a subwavelength optical fiber. Laser Photonics Rev..

[47] Harada Y., Asakura T. (1996). Radiation forces on a dielectric sphere in the Rayleigh scattering regime. Opt. Commun..

[48] Ribeiro R.S.R., Soppera O., Oliva A.G., Guerreiro A., Jorge P.A.S. (2015). New trends on optical fiber tweezers. J. Lightwave Technol..

[49] Gong Y., Ye A.Y., Wu Y., Rao Y.J., Yao Y., Xiao S. (2013). Graded-index fiber tip optical tweezers: Numerical simulation and trapping experiment. Opt. Express.

[50] Nylk J., Kristensen M.V.G., Mazilu M., Thayil A.K., Mitchell C.A., Campbell E.C., Dholakia K. (2015). Development of a graded index microlens based fiber optical trap and its characterization using principal component analysis. Biomed. Opt. Express.

[51] Gordon R., Kawano M., Blakely J.T., Sinton D. (2008). Optohydro dynamic theory of particles in a dual-beam optical trap. Phys. Rev. B.

[52] Liu Y., Yu M. (2009). Investigation of inclined dual-fiber optical tweezers for 3D manipulation and force sensing. Opt. Express.

[53] Huang J., Liu X., Zhang Y., Li B. (2015). Optical trapping and orientation of Escherichia coli cells using two tapered fiber probes. Photonics Res..

[54] Liu X., Huang J., Zhang Y., Li B. (2015). Optical regulation of cell chain. Sci. Rep..

[55] Zhang Y., Liu Z., Yang J. (2012). A non-contact single optical fiber multi-optical tweezers probe: Design and fabrication. Opt. Commun..

[56] Zhao H., Farrell G., Wang P. (2016). Investigation of particle harmonic oscillation using four-core fiber integrated twin-tweezers. IEEE Photonics Technol. Lett..

[57] Deng H., Zhang Y., Yuan T. (2017). Fiber-based optical gun for particle shooting. ACS Photonics.

[58] Liberale C., Minzioni P., Bragheri F., Angelis F.D., Fabrizio E.D., Cristiani I. (2007). Miniaturized all-fibre probe for three-dimensional optical trapping and manipulation. Nat. Photonics.

[59] Unterkofler S., Garbos M.K., Euser T.G., Russell P.S.J. (2013). Long-distance laser propulsion and deformation-monitoring of cells in optofluidic photonic crystal fiber. J. Biophotonics.

[60] Gong Y., Zhang C., Liu Q.F., Wu Y., Wu H., Rao Y., Peng G.D. (2015). Optofluidic tunable manipulation of microparticles by integrating graded-index fiber taper with a microcavity. Opt. Express.

[61] Zhang C.L., Gong Y., Liu Q.F., Wu Y., Rao Y.J., Peng G.D. (2016). Graded-index fiber enabled strain-controllable optofluidic manipulation. IEEE Photonics Technol. Lett..

[62] Gong Y., Liu Q.F., Zhang C.L., Wu Y., Rao Y.J., Peng G.D. (2015). Microfluidic flow rate detection with a large dynamic range by optical manipulation. IEEE Photonics Technol. Lett..

[63] Gong Y., Qiu L., Zhang C., Wu Y., Rao Y.J., Peng G.D. (2017). Dual-Mode fiber optofluidic flowmeter with a large dynamic range. J. Lightwave Technol..

[64] Xin H., Zhang Y., Lei H., Li Y., Zhang H., Li B. (2013). Optofluidic realization and retaining of cell–cell contact using an abrupt tapered optical fibre. Sci. Rep..

[65] Liu X., Huang J., Li Y., Zhang Y., Li B. (2017). Optofluidic organization and transport of cell chain. J. Biophotonics.

[66] Zhang Y., Li Y., Zhang Y., Hu C., Liu Z., Yang X., Yuan L. (2018). HACF-based optical tweezers available for living cells manipulating and sterile transporting. Opt. Commun..

[67] Xin H., Li B. (2019). Fiber-based optical trapping and manipulation. Front. Optoelectron..

[68] Xu C., Lei H., Zhang Y., Li B. (2012). Backward transport of nanoparticles in fluidic flow. Opt. Express.

[69] Lei H., Zhang Y., Li B. (2012). Particle separation in fluidic flow by optical fiber. Opt. Express.

[70] Gloge D. (1971). Weakly guiding fibers. Appl. Opt..

[71] Li K., Liu G., Wu Y., Hao P., Zhou W., Zhang Z. (2014). Gold nanoparticle amplified optical microfiber evanescent wave absorption biosensor for cancer biomarker detection in serum. Talanta.

[72] Sun D., Guo T., Ran Y., Huang Y., Guan B. (2014). In situ DNA hybridization detection with a reflective microfiber grating biosensor. Biosens. Bioelectron..

[73] Soong C., Li W., Liu C., Tzeng P. (2010). Theoretical analysis for photophoresis of a microscale hydrophobic particle in liquids. Opt. Express.

[74] Duhr S., Braun D. (2006). Optothermal molecule trapping by opposing fluid flow with thermophoretic drift. Phys. Rev. Lett..

[75] Xin H., Li X., Li B. (2011). Massive photothermal trapping and migration of particles by a tapered optical fiber. Opt. Express.

[76] Lei H., Zhang Y., Li X., Li B. (2011). Photophoretic assembly and migration of dielectric particles and Escherichia coli in liquids using a subwavelength diameter optical fiber. Lab. Chip.

[77] Xin H., Lei H., Zhang Y., Li X., Li B. (2011). Photothermal trapping of dielectric particles by optical fiber-ring. Opt. Express.

[78] Cadarso V.J., Llobera A., Puyol M. (2015). Integrated photonic nanofences: Combining subwavelength waveguides with an enhanced evanescent field for sensing applications. ACS Nano.

[79] Gong Y., Zhang M., Gong C. (2015). Sensitive optofluidic flow rate sensor based on laser heating and microring resonator. Microfluid. Nanofluid..

[80] Liu Z., Lei J., Zhang Y. (2015). A Method to Gather/Arrange Particles Based on Thermal Convection. Proceedings of the 24th International Conference on Optical Fibre Sensors.

[81] Li Z., Yang J., Liu S., Jiang X., Wang H., Hu X., Xing X. (2018). High throughput trapping and arrangement of biological cells using self-assembled optical tweezer. Opt. Express.

[82] Yang J., Li Z., Wang H., Zhu D., Cai X., Cheng Y., Xing X. (2017). Optofluidic trapping and delivery of massive mesoscopic matters using mobile vortex array. Appl. Phys. Lett..

[83] Zhang C.L., Gong Y., Zou W.L., Wu Y., Rao Y.J., Peng G.D., Fan X. (2017). Microbubble-Based Fiber Optofluidic Interferometer for Sensing. J. Lightwave Technol..

[84] Zhang C.L., Gong Y., Wu Y., Rao Y., Peng G., Fan X. (2018). Lab-on-tip based on photothermal microbubble generation for concentration detection. Sens. Actuators B.

[85] Matjasec Z., Donlagic D. (2017). All-optical, all-fiber, thermal conductivity sensor for identification and characterization of fluids. Sens. Actuators B.

[86] Zhang K., Jian A., Zhang X., Wang Y., Li Z., Tam H.Y. (2011). Laser-induced thermal bubbles for microfluidic applications. Lab. Chip.

[87] Tovar A.R., Patel M.V., Lee A.P. (2011). Lateral air cavities for microfluidic pumping with the use of acoustic energy. Microfluid. Nanofluid..

[88] Ahmed D., Chan C.Y., Lin S.C.S., Muddana H.S., Nama N., Benkovic S.J., Huang T.J. (2013). Tunable pulsatile chemical gradient generation via acoustically driven oscillating bubbles. Lab. Chip.

[89] Rogers P., Neild A. (2011). Selective particle trapping using an oscillating microbubble. Lab. Chip.

[90] Xu Y., Hashmi A., Yu G., Lu X., Kwon H.J., Chen X., Xu J. (2013). Microbubble array for on-chip worm processing. Appl. Phys. Lett..

[91] Fujii S., Kobayashi K., Kanaizuka K., Okamoto T., Toyabe S., Muneyuki E., Haga M.A. (2009). Manipulation of single DNA using a micronanobubble formed by local laser heating on a Au-coated surface. Chem. Lett..

[92] Davim J.P. (2013). Nontraditional Machining Processes: Research Advances.

[93] Cheng Y., Yang J., Li Z., Zhu D., Cai X., Hu X., Xing X. (2017). Microbubble-assisted optofluidic control using a photothermal waveguide. Appl. Phys. Lett..

[94] Skirtach A.G., Munoz Javier A., Kreft O., Köhler K., Piera Alberola A., Möhwald H., Sukhorukov G.B. (2006). Laser-induced release of encapsulated materials inside living cells. Angew. Chem. Int. Ed..

[95] Ochs M., Carregal-Romero S., Rejman J., Braeckmans K., De Smedt S.C., Parak W.J. (2013). Light-Addressable Capsules as Caged Compound Matrix for Controlled Triggering of Cytosolic Reactions. Angew. Chem. Int. Ed..

[96] Dykman L., Khlebtsov N. (2012). Gold nanoparticles in biomedical applications: Recent advances and perspectives. Chem. Soc. Rev..

[97] Loo C., Lowery A., Halas N., West J., Drezek R. (2005). Immunotargeted nanoshells for integrated cancer imaging and therapy. Nano Lett..

[98] Kim Y.K., Na H.K., Kim S., Jang H., Chang S.J., Min D.H. (2015). One-Pot Synthesis of Multifunctional Au@ Graphene Oxide Nanocolloid Core@ Shell Nanoparticles for Raman Bioimaging, Photothermal, and Photodynamic Therapy. Small.

[99] Shao J., Xuan M., Dai L., Si T., Li J., He Q. (2015). Near-Infrared-Activated Nanocalorifiers in Microcapsules: Vapor Bubble Generation for In Vivo Enhanced Cancer Therapy. Angew. Chem. Int. Ed..

[100] Gong C., Gong Y., Chen Q., Rao Y.J., Peng G.D., Fan X. (2017). Reproducible fiber optofluidic laser for disposable and array applications. Lab Chip.

[101] Yang X., Shu W., Wang Y., Gong Y., Gong C., Chen Q., Rao Y.J. (2019). Turbidimetric inhibition immunoassay revisited to enhance its sensitivity via an optofluidic laser. Biosens. Bioelectron..

